# Chloride Removal of Calcium Aluminate-Layered Double Hydroxide Phases: A Review

**DOI:** 10.3390/ijerph18062797

**Published:** 2021-03-10

**Authors:** Gwangmok Kim, Sangwon Park

**Affiliations:** Center for Carbon Mineralization, Mineral Resources Division, Korea Institute of Geoscience and Mineral Resources 124 Gwahak-ro, Yuesong-gu, Daejeon 34132, Korea; k.gm@kigam.re.kr

**Keywords:** CaAl-LDH phases, chloride removal, positively charged interlayers, chemisorption, removal rate and capacity

## Abstract

Chlorine is a critical element with respect to the use of fossil fuel, recycling of industrial wastes, and water purification. Chlorine could form toxic chemical compounds, corrode pipe systems and boilers, and contaminate surface and ground waters. Calcium aluminate-layered double hydroxides are one of the most promising materials to remove chlorides due to the chemisorption mechanism, since the phases have positively charged interlayers. Many studies on the synthesis and the characterization of calcium aluminate-layered double hydroxides have been extensively conducted, whereas few studies have been conducted on the chloride removal characteristics of the phases. The state-of-the-art studies on the synthesis methods and the structural characteristics of CaAl-LDH phases, the underlying mechanism on the removal of chlorides, and the potential removal rate and the capacity in the present study were thoroughly reviewed.

## 1. Introduction

Chlorine has been widely adopted for disinfection in the urban water supply [1]. The use of chlorine restrains the propagation of viruses, and pathogenic bacteria and organisms [2]. Residual chlorine in water may kill microorganisms [2]. However, surplus chlorine may negatively affect the taste of water and form trihalomethanes which can cause cancer [3,4]. The World Health Organization (WHO) has provided the guidelines in which the concentration of the residual free chlorides in pipes should be limited in the range of 0.03–0.4 mg/L [5].

Various fuel sources and industrial wastes such as coal gangue and municipal incineration solid wastes generated from the paper industry, polyvinylchlorides, and household wastes also include chlorine [6,7,8,9,10,11]. The chlorine during combustion can corrode plumbing pipes and boilers, and lead to the generation of acid rain [12]. The chlorine leached from the sources and wastes may contaminate surface and ground waters, and soils [13]. The chlorine included in the sources and wastes thus inhibits the effective use and increases the cost of the process.

Chlorides are one of the critical elements with respect to the durability of cementitious materials. High alkaline condition (pH ≈ 12) generated from cementitious materials induces a passive state of reinforcing bars so that corrosion of the bars is inhibited [14]. The presence of excessive chloride contents in cementitious materials leads to the breakdown of the passive state on the surface of reinforcing bars, which in turn initiates corrosion [14]. The control over chloride contents in cementitious materials has been an important issue when the materials are exposed to marine conditions or de-icing salts [15].

Therefore, various methods for the removal of chlorides such as activated carbon adsorption, redox processes with filter medium, water extraction, hydrotalcites and thermal treatment have been attempted [9,10,11,16,17,18]. Water extraction is a representative method for the removal of chlorides. This method is effective at removing chloride salts on the surface of a matter [17]. However, this method could generate wastewater including a large amount of chlorides, and lead to the leaching of other heavy metal ions [17]. Thermal treatment conditions (above 900 °C) can evaporate the chlorides, but the treatment method is not cost-effective and increases environmental loads [18]. In this context, the adsorption process with various absorbents has been adopted, owing to the simple operation and cost-effectiveness [16]. Iron oxides and hydroxides, titanium, and some composites with iron and titanium have been often used as an adsorbent [16,19].

Calcium aluminate-layered double hydroxides (CaAl-LDH) phases are capable of removing chlorides induced by chemisorption mechanism; thus, the phases have potential applicability as an adsorbent. The structural characteristics, chloride removal mechanism, synthesis methods, and potential chloride removal rate and capacity of CaAl-LDH phases in the present study were thoroughly reviewed. The present paper provides in-depth information to adopt CaAl-LDH phases as an adsorbent for the removal of chlorides.

## 2. Calcium Aluminate-Layered Double Hydroxide Phases

### 2.1. Structure

The general formula of LDHs is [M^2+^, M^3+^(OH)_2x+2_] + [A_1/n_^−1^·*m*H_2_O]^−^. Here, M^2+^ and M^3+^ represent cations being divalent and trivalent, respectively, while A represents an n-valent anion. The LDHs have characteristic structures that are positively charged (M^2+^, M^3+^) octahedral layers [20]. The M^2+^ can be substituted with M^3+^ so that the space between the interlayers is positively charged. Thus, the interlayer could include the anions and water molecules [20].

Calcium aluminate-LDH phases are one of the crystalline minerals, mainly consisting of a calcium-aluminate system [21]. The main layer is lamellar portlandite-like structures consisting of the ordered arrangement of Ca^2+^ and Al^3+^ ions [21]. Rousselot et al. [20] reported that the interlayer distance of CaAl-LDH phases was approximately 5.81 Å. The interlayer distance can be varied with the M^2+^/M^3+^ ratios [22]. The interlayer distance decreases when the ratio of Ca/Al is low, since the electrostatic attraction force between positive and negative layers increases [22]. The Al^3+^ could be replaced by Cr^3+^. Ga^3+^, Fe^3+^, while Ca^2+^ could be replaced by Cd^2+^ [23,24,25]. The replacement of trivalent cations in the phases also could change the interlayer distance [26].

### 2.2. Formation

The synthesis of CaAl-LDH phases could be affected by various factors such as the species variation of main components, thermodynamic characteristics of starting materials, and reaction routes [19,27,28,29]. The various factors are determined by the type of synthesis method, temperature, anion, and pH value and so on. The effect of the variables on the synthesis of CaAl-LDH phases are described in the following section.

#### 2.2.1. Methods for Synthesis

Table 1 showed the summary of synthesis methods for CaAl-LDH phases in previous studies [20,27,30,31,32,33]. The coprecipitation method is the reaction process slowly adding di- and trivalent metal salts to an alkaline solution, which is often used for the synthesis of CaAl-LDH phases [20,30,34,35]. Two types of starting materials for the supply of calcium and aluminum sources are generally used. The most representative calcium source is calcium nitrate tetrahydrate (Ca(NO_3_)_2_·4H_2_O), whereas an aluminum source is aluminum nitrate nonahydrate (Al(NO_3_)_3_·9H_2_O) [20,30,34,35]. The final product is CaAl-NO_3_ LDH phases [27]. The procedures of the method are briefly as follows [20,27,30,33,35]; Ca and Al sources are dissolved in deionized (DI) water. In most cases, the Ca/Al ratio is fixed at 2:1, since the stoichiometric ratio of the CaAl-LDH phases is identical [30]. The mixed solution is poured into sodium nitrate and stirred with a magnetic stirrer for 2.0–3.5 h at 25–65 °C [20,30,35]. The pH value of the solution is titrated by sodium hydroxide (NaOH). The designated pH values in previous studies were in the range of 10.0–12.5 [20,30,35]. Then, the precipitated particles are filtered and washed with DI water until the soluble nitrates are mostly removed. The washed product is aged at 60–160 °C for 12–24 h and dried.

Ultra-high lime with aluminate process (UHLA) method was also used for the synthesis of CaAl-LDH phases. Calcium hydroxide (Ca(OH)_2_) and sodium aluminate (NaAlO_2_) as starting materials were generally used when applying the method in the previous studies [31,36]. This is a modification method of lime softening that is used in the fields for the removal of leading scalants such as Ca^2+^, Mg^2+^, and SiO_2_ [36]. The key factor of this process is to ensure high pH (11.0–12.0) and calcium conditions by supplying lime [36]. Calcium aluminate-LDH phases can be synthesized by mixing starting materials for a few h at 25–40 °C. Calcium and aluminum sources are mixed and stirred at 40 °C for 3.5 h.

Furthermore, various methods for the synthesis of CaAl-LDH phases have been successfully attempted. Szabados et al. [37] utilized mechanical milling and ultrasound to synthesis CaAl-LDH phases with portlandite and gibbsite (Al(OH)_3_).

#### 2.2.2. Anions

Anions included in starting materials when synthesizing CaAl-LDH phases could act as a stabilizer of the structure. Szabados et al. [37] reported in the previous study that the added carbonate (CO_3_^2−^) became interlayer ions when the concentration was lower than 0.1 M, and prohibited the transformation of CaAl-LDH phases to hydrated tricalcium aluminate. When increasing the concentration of carbonate, the basal spacing decreased, forming LDH phases with monocarbonates from those with hemicarbonates [37]. However, the calcium carbonates were dominantly formed, when the concentration of carbonate ions reached more than 1.0 M [37]. Chlorine, bromide and iodide ions as stabilizers can be also utilized for the synthesis of the CaAl-LDH phases [38]. In general, the affinity order of anions in the interlayer of CaAl-LDH phases is as follows: F^−^ > Cl^−^ ≈ CO_3_^2−^ > Br^−^ > I^−^, meaning that the intercalation of the anions when forming the CaAl-LDH phases could prevent the transformation into other phases [30].

#### 2.2.3. pH Value

**Figure 1:**
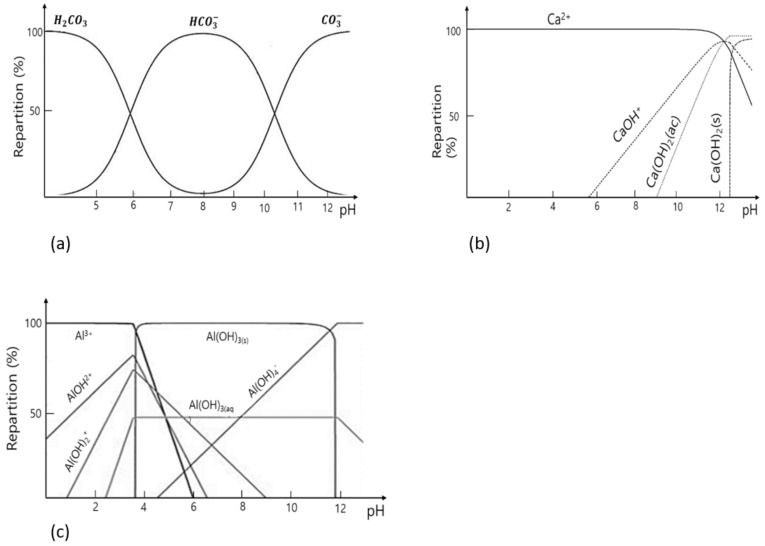
Variation of (**a**) carbonate, (**b**) calcium, and (**c**) aluminum species with pH [39,43,46,47].

The pH condition of solutions with starting materials for the synthesis of CaAL-LDH phases is a critical factor, since the concentrations of H+ and OH- in the solutions significantly affect the yield of the CaAl-LDH phases and the formation of byproducts. The CaAl-LDH phases in previous studies were generally synthesized at a pH in the range of 10.0–12.5 [20,30,33]. Ca^2+^, Ca(OH)+ and Ca(OH)_2_ at the pH condition are dominant species, while Al(OH)_3_(s), Al(OH)_3_(aq) and Al(OH)_4_^−^ ion are dominant species of Al hydrolysis (see Figure 1b,c). According to [31], the reaction routes for the formation of CaAl-LDH phases at the pH conditions may be mainly as follows.

4Ca^2+^ + 2Al(OH)_4_^−^ + (2/n)·*R*^n−^ + 4OH^−^ ↔ Ca_4_Al_2_O_6_((2/n)·*R*)·10H_2_O (1)

3Ca(OH)_2_ + 2Al(OH)_3_ ↔ Ca_3_Al_2_(OH)_12_ (2)

Ca_3_Al_2_(OH)_12_ + Ca(OH)_2_ + 6H_2_O ↔ 2Ca_2_Al(OH)_7_·3H_2_O (3)

where *R*^n^^−^ represent interlayer anions such as OH^−^, Cl^−^, NO_3_^2−^, CO_3_^2−^ and etc. Equation (1) could be the dominant reaction route at a pH value in the range of 8.0–10.0, while the combination of Equations (2) and (3) are reaction route at a pH value in the range of more than 10.0. There are three types of carbonate ions depending on pH conditions (see Figure 1a) [39]. The CO_3_^2−^ species sharply increases when the pH value is more than 10.0. The ionic charge of calcium ions in solutions under this condition is identical to that of carbonate ions (see Figure 1b). Furthermore, the ionic radii of calcium (100 pm) and carbonate (178 pm) ions are almost similar [40]. Thus, polymorphs of calcium carbonates were readily precipitated under this condition. Xu et al. [41] reported that an increase in the pH values more than 12.0 led to the formation of Ca(OH)_2_ and Al(OH)_3_, and the formation of CaCO_3_ was observed at a pH of 10.5–12.5. Analogous phenomena with respect to the formation of CaCO_3_ in water solution systems have been reported in previous studies [42,43]. In this regard, Saha et al. [44] investigated the effect of pH conditions on the synthesis of CaAl-LDH phases and reported that CaAl-LDH phases with a purity of 100% were synthesized at a pH of 8.5. The purity of CaAl-LDH phases became lower as the pH values increased, leading to the formation of calcium carbonates such as calcites and aragonites.

Overall, it could be inferred from previous studies that a proper pH value for the synthesis of CaAl-LDH phases is approximately 8.0–10.0. Xu et al. [41] proposed the use of ethanol to prevent the formation of CaCO_3_ during the synthesis of CaAl-LDH phases. Iyi et al. [45] also reported that the use of ethanol could prevent intercalate carbonate ions to LDH phases. It was attributable that the solubility of carbon dioxide in solutions with ethanol was remarkably low.

#### 2.2.4. Temperature

Szabados et al. [37] reported that the yield of CaAl-LDH phases dramatically increased when the temperature was more than 40 °C. Xu et al. [41] reported that the intensity of the characteristic peak for CaAl-LDH phases in XRD patterns increased when the temperature for the synthesis was from 70–130 °C. It is well known that the dehydration of CaAl-LDH phases is endothermic and the temperatures at which the reaction occurs are approximately 100 °C, 275 °C, and 550 °C [44]. The dehydration reaction at approximately 100 °C is caused by the desorption of the physisorbed and interlayer water of CaAl-LDH phases [48]. The dehydration characteristics at about 100 °C are possibly responsible for the proper temperature limit for the synthesizing CaAl-LDH phases. Overall, it can be inferred from the previous studies on the effect of temperature on the synthesis of CaAl-LDH phases that a proper temperature for the synthesis is possibly in the range of 40–100 °C [37,41,48].

Meanwhile, the dissolution reaction characteristics of starting materials could affect the temperature for synthesizing CaAl-LDH phases. An increase in the temperature could affect the synthesis of CaAl-LDH phases when the starting materials exhibiting exothermic dissolution behavior are used. For instance, the solubility of Ca(OH)_2_ and CaCl_2_, which are dissolved by the exothermic reaction process, is reduced as the temperature for the synthesis of CaAl-LDH phases increases. Szabados et al. [37] reported that CaAl-LDH phases were formed by a solid-state reaction due to the exothermic dissolution behavior of Ca(OH)_2_ at a temperature of more than 40 °C, and the condition promoted the transformation of the LDH phases into tricalcium-aluminate phases. That is, the temperature for synthesizing CaAl-LDH phases could be varied with the dissolution characteristics of starting materials.

#### 2.2.5. Tricalcium Aluminate Precursors

Tricalcium aluminate (C_3_A) phases are one of the components of ordinary Portland cement [49]. The C_3_A phases can be formed by the incineration of CaAl-LDH phases. Calcium aluminate-LDH phases in the temperature range from 800–900 °C were reportedly transformed into C_12_A_7_ (12CaO·7Al_2_O_3_) and C_3_A (3CaO·Al_2_O_3_) phases [48,50,51]. The C_3_A phases during the hydration reaction process of cementitious materials react with gypsum (CaSO_4_·2H_2_O) and mainly form ettringite (Ca_6_Al_2_(SO_4_)_3_(OH)_12_·26H_2_O), while the phases exposed to chloride conditions can be transformed into Cl-bearing CaAl-LDH phases [52,53,54,55]. The hydration characteristics of C_3_A in previous studies have been employed to remove chlorides in cementitious materials, since the chlorides can lead to corrosion of the rebars in cementitious materials [30,35,56,57,58].

### 2.3. Chloride Removal Mechanism

The chloride removal of CaAl-LDH phases is induced by the structural characteristic. The CaAl-LDH phases consist of lamellar portlandite-like layers with an arrangement of Ca^2+^ of octahedral coordination and Al^3+^ of heptahedral coordination [20]. The LDH phases have thus layered structures consisting of positively charged sheets due to the periodical stacking of Ca^2+^ and Al^3+^ ions and interlayer spaces [43,59]. The interlayer spaces could contain solvated anions or water molecules due to the charge imbalance between the sheets and interlayer spaces [60]. The intercalation of the anions or water molecules to the interlayers causes the electrically neutral state of the CaAl-LDH structures [20]. Palin et al. [61] reported that the anion intercalation process initiated at 2D interface, advancing one direction. The anions entered the interlayers and the intercalation occurred along the layers in the 1D dimension. The reaction process reportedly occurred within tens of seconds and was limited by diffusion of the anions [61]. Various anions in the interlayer spaces are intercalated with hydrogen bonding, meaning that the anions may be exchanged with other anions due to the affinity orders [37,62]. Chlorine ions are one of the anions capable of intercalating into the interlayers of CaAl-LDH phases. As aforementioned, the affinity order of the anions in the interlayer of the phases is F^−^ > Cl^−^ ≈ CO_3_^2−^ > Br^−^ > I^−^ [30]. The affinity order is mainly affected by the valence states and ionic radii of the anions [30]. Overall, CaAl-LDH phases can remove chlorine ions by chemisorption mechanism, since the phases provide sites for anions as interlayer spaces and electrical attraction forces due to the charge imbalance. The intercalation of chloride ions could be mainly affected by the affinity order and the diffusion characteristics.

## 3. Estimation of Binding Capacity and Adsorption Rate

### 3.1. Kinetic Models and Adsorption Rate

The potential adsorption rate of absorbents has been generally estimated by pseudo-first-order, pseudo-second-order, Elovich kinetic models [63,64]. The major difference among the models is the adsorption mechanism. Pseudo-first-order kinetic model is applicable when physisorption is dominant, while pseudo-second-order and Elovich kinetic model showed a good fit when chemisorption is dominant. The equations of pseudo-first-order, pseudo-second-order, and Elovich kinetic models in order are as follows, respectively.

*ln*(*Q_e_* − *Q_t_*) = *ln*(*Q_e_* − *k*_1_·*t*) (4)

*t*/*Q_t_* = 1/(*k*_2_*Q_e_*^2^) + *t*/*Q*_1_ (5)

*Q_t_* = *ln*(*αβ*)/*β* + *ln*(*t*)/*β* (6)

where *Q_t_* (mg/g) and *Q_e_* (mg/g) represent the concentration of bound chlorides on CaAl- LDH phases at time *t* and equilibrium state, respectively. *k*_1_ and *k*_2_ indicate rate constant and specific rate constant of each kinetic model, respectively [65]. *k*_2_*Q_e_*^2^ (mg·g^−1^·h^−1^) indicates the initial chemisorption rate constant in the pseudo-second-order model [66]. *α* and *β* in the Elovich kinetic model is the initial sorption rate constant (mg/g days) and the desorption constant (g/mg), respectively.

The underlying mechanism of chloride removal of CaAl-LDH phases was reportedly chemisorption. Thus, the pseudo-second-order kinetic model was generally used in previous studies on the chloride removal of CaAl-LDH phases [31,67,68,69]. Yoon et al. [69] compared the fit of three models and reported that the pseudo-second-order kinetic model showed a good fit with the results.

There are few available data on the potential chloride removal rate of CaAl-LDH phases. Table 2 showed the potential chloride removal rate of CaAl-LDH phases obtained by the pseudo-second-order kinetic model in previous studies [31,67,68]. The values of *k*_2_ indicating specific rate constant were in the range of 0.0019–0.0046 g·mg^−1^·min^−1^. In particular, the values of *k*_2_ in the previous study increased by 0.009 g·mg^−1^·min^−1^ when Fe ions were present as a form of CaFeAl-NO_3_ LDH phases, meaning that the removal rate of CaFeAl-NO_3_ LDH phases was improved compared to CaAl-NO_3_ LDH phases [67]. Yang et al. [67] reported that the addition of Fe increased the specific surface area and total pore volume of CaAl-LDH phases, and thereby the chemisorption capability of chlorides was improved. The chloride removal induced by chemisorption of CaAl-LDH phases occurred within a few hours. Chi et al. [31] reported that as much as 40% of chloride contents within 10 min was removed by CaAl-LDH phases and the calculated chloride removal capacity by means of pseudo-second order kinetic model in the previous study was in the range from 23.1–25.7 mg·g^−1^ [31]. It was also reported in previous studies conducted by Zhang et al. [68] that more than 90% of chloride content within 4 h was removed by CaAl-LDH phases and the calculated chloride removal capacity by means of pseudo-second order kinetic model in the previous study was 91.844 mg·g^−1^. Meanwhile, the difference in a value of *k*_2_*Q_e_*^2^ in previous studies was large [31,68]. The value reported in [31] was 1.58–1.64 mg·g^−1^·h^−1^, while that reported in [68] was 10.038 mg·g^−1^·h^−1^. The difference was induced by the value of *Q_e_*. The *Q_e_* value reported in [68] was 10 times greater than that reported in [31]. A further study is needed to investigate the estimation of reasonable initial chemisorption rate constant, since there are few available data.

### 3.2. Isotherm Models of Chloride Removal

**Figure 2:**
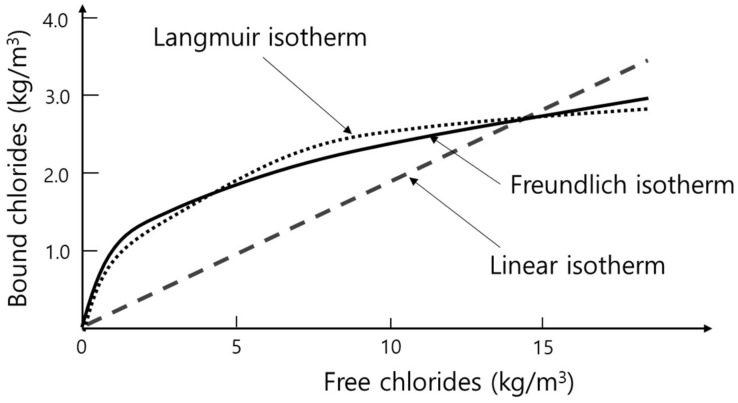
Comparison of Linear, Langmuir and Freundlich isotherm models [72].

Isotherm model is the relationship between free and bound ligand at a given temperature [70]. That is, the isotherm indicates the potential removal capacity of an absorbent as a function of free ligands. There are three types of isotherm models on the chloride removal generally used in previous studies [30,67,68]. Linear isotherm proposed by [71] is the most simplified method and expressed as follows,

*C_b_* = *kC_f_* (7)

where *C_b_* and *C_f_* represent the concentration of bound and free chlorides, respectively. *k* indicates a constant. It is generally accepted that the linear isotherm is effective when the concentration of free chlorides is lower than 20,000 ppm [15]. As shown in Figure 2, the linear binding isotherm overestimates bound chlorides at a high concentration of free chlorides, while the isotherm underestimates bound chlorides at a low concentration [72].

Langmuir and Freundlich models derived from physical chemistry are representative non-linear isotherms [73,74]. Langmuir isotherm assumed monolayer adsorption, meaning that this model in previous studies has been an excellent fit for absorbents with homogenous adsorption sites [30,75,76,77]. Chi et al. [31] reported that the Langmuir model was an excellent fit for the synthesized CaAl-LDH phases when removing chlorides, since the underlying mechanism of the removal was chemisorption exchanging anions in the interlayer of the phases. The isotherm model can be expressed as [78]:

*C_b_* = *K_L_Q_m_C_f_*/(1 + *K_L_C_f_*) (8)

where *C_b_* indicates the concentration of bound chlorides on the outer surface of an absorbent, while *Q_m_* represents maximum adsorption content of the chlorides by absorbent, i.e., indicating the binding capacity of the absorbent. *C_f_* and *K_L_* indicate the concentration of free chlorides and the constant of Langmuir isotherm, respectively. The equation indicates that the slope of the isotherm curve is converged to zero when the concentration of free chlorides is high, since the bound chlorides reach the value of *Q_m_*.

Freundlich isotherm is applicable when the concentration is high or the adsorption sites of an absorbent are heterogeneous. Luping and Nilsson reported that monolayer adsorption was generated when the concentration of free chlorides was low (≈0.05 M), while the adsorption mechanism was more complicated when the concentration of free chlorides was high [79]. Thus, this model has shown a good fit in the previous studies on the chloride removal of the cementitious system under seawater conditions [80,81]. It is well known that the concentration of chlorides in seawater is approximately 20,000 ppm, meaning a high concentration condition [82]. Meanwhile, various hydration products such as calcium silicate hydrates formed by C_3_S and C_2_S, and Cl-bearing CaAl-LDH phases formed by C_3_A in cementitious systems can act as an absorbent of chlorides. In particular, the C_3_A precursor removed the chlorides by occlusion, inclusion and adsorption, forming CaAl-LDH at a high concentration of chlorides [83]. The inclusion of chlorides occurs when forming CaAl-LDH-Cl and leads to the formation of defects in the crystal lattice, while the occlusion occurs in chlorides weakly bound to the surface when the crystal growth of CaAl-LDH-Cl is progressed [84]. Adsorption occurs due to charge imbalance in the interlayer of CaAl-LDH phases [84].

### 3.3. Removal Capacity

The potential chloride removal capacity of CaAl-LDH phases has been mainly investigated in the field of cementitious materials, since the penetration of chlorides into cementitious matrix causes the corrosion of reinforcing bar in concrete structures [85].

Table 3 showed the potential chloride removal capacity of CaAl-LDH phases in previous studies [30,67,68]. The potential chloride removal capacity of CaAl-LDH phases has mostly been investigated via the Langmuir isotherm model, since the underlying mechanism of Cl binding was reportedly caused by chemisorption on homogenous sites in the interlayers of CaAl-LDH phases [30]. The theoretical chloride removal capacity of CaAl-LDH phases with respect to stoichiometry is approximately 130–140 mg/g [30]. The experimentally estimated potential chloride removal capacity via the Langmuir isotherm model was in the range of 118.59–211.324 mg/g [30,67,68]. However, further studies on the chloride removal capacity of CaAl-LDH phases should be conducted to clearly verify the value of chloride removal capacity reported in the previous studies, since available data on the chloride removal capacity of CaAl-LDH phases were few [30,67,68].

Meanwhile, the overestimated chloride removal capacity of CaAl-LDH phases in the previous study conducted by Chen et al. [30] was probably induced by the other hydrates when adding CaAl-LDH phases to a cementitious matrix. Various hydrates including C-S-H and CaAl-LDH phases in a cementitious matrix could contribute to the chloride removal. For instance, C-S-H phases could adsorb chlorine ions due to the chemisorption on the layer of phases, the penetration into the interlayer space, and encapsulation in the C-S-H lattice. Meanwhile, the anion affinity order of the interlayer in CaAl-LDH phases is one of the most significant factors affecting the chloride removal capacity. Zhang et al. [68] reported that the chloride removal capacity of CaAl-LDH phases was significantly reduced when CO_3_^2−^ and SO_4_^2−^ ions were present, while that of the phases was almost similar when OH^−^ ion was present.

## 4. Conclusions

Residual chlorine generated from industrial sectors related to the urban water supply system and utilization of coal gangue and municipal incineration solid wastes can cause the formation of trihalomethanes, a kind of carcinogen, and generate acid rain. In this context, the removal of chlorine is critical for public health. Calcium aluminate-LDH phases have the capability to remove chlorides induced by chemisorption; thus, the phases have potential applicability as an adsorbent. The structural characteristics, chloride removal mechanism, synthesis methods, and potential chloride removal rate and capacity of CaAl-LDH phases in the present study were thoroughly reviewed and thus provided in-depth information to adopt CaAl-LDH phases as an adsorbent for the removal of chlorides. The following concluding remarks are drawn from the present study.

(1) The CaAl-LDH phases have unique structural characteristics induced by the interlayers similar to lamellar portlandite-like structures. The interlayers consist of cations being divalent and trivalent, which is responsible for the positive charge between the interlayers. The positively charged interlayers can include the anions and water molecules. The understanding of the structural characteristics is helpful for the application of CaAl-LDH phases as an adsorbent.
(2) Various synthesis techniques such as coprecipitation, ultra-high lime with the aluminate process, and green synthesis methods have been adopted to synthesize CaAl-LDH phases. Factors including temperature, anions provided by starting materials, pH values, and so on significantly affected the formation of CaAl-LDH phases. Proper conditions for the synthesis of CaAl-LDH phases in the present study were summarized.
(3) The underlying mechanism for the chloride removal of CaAl-LDH phases was introduced. The chemisorption due to the positively charged interlayers in the phases mainly contributes to the removal of chlorides. The presence of exchangeable anions also significantly affected the removal of chlorides, owing to affinity orders.
(4) Representative kinetic and isotherm models were introduced and summarized results reported in previous studies to estimate the potential chloride removal rate and capacity of CaAl-LDH phases.

Overall, CaAl-LDH phases exhibited potential applicability as an adsorbent for the removal of chlorides from aqueous media, considering the potential removal rate and capacity. However, further studies are needed to deeply investigate the initial adsorption rate desorption characteristics of chlorides in CaAl-LDH phases based on structural characteristics.

## Figures and Tables

**Table 1:** Summary of synthesis methods for CaAl-LDH phases in previous studies [20,27,30,31,32,33].

Starting Materials		Condition		Aging Condition		Ca/Al Molar Ratio	Final Product	Synthesis Method	Ref.
Cation	Anion	Temp. (°C)	pH	Period (h)	Temp. (°C)				
Ca(NO_3_)_2_·4H_2_O	Al(NO_3_)_3_·9H_2_O	25	12	-	-	2.0:1	Ca-Al-NO_3_	Coprecipitation method	[30]
CaCl_2_	M^3+^Cl_3_ or M^3+^(NO_3_)_3_	>65	11.5 ± 0.1	24	35	-	Ca-M^3^-Cl	Coprecipitation method	[20]
Ca(OH)_2_	NaAlO_2_	40	12.65–12.78	-	-	2.0:1	Ca-Al-OH	Ultra-high lime with Aluminate process method	[31]
CaO·Al_2_O_3_	CaCrO_4_	25	7, 10, 12	-	-	5.08:2.0	Ca-Al(Cr)	-	[32]
Ca(NO_3_)_2_·4H_2_O	Al(NO_3_)_3_·9H_2_O	25	10	24	160	3.0:1	Ca-Al-NO_3_	Coprecipitation method	[27]
Ca(NO_3_)_2_·4H_2_O	Al(NO_3_)_3_·9H_2_O	25	12.5	12	60	2.0:1	Ca-Al-NO_3_	Coprecipitation method	[33]
Ca(OH)_2_	γ-AlO(OH)	25	12.5	6	80	2.0:1	Ca-Al-OH	Green synthesis method	

M^3+^: Al^3+^, Ga^3+^, Fe^3+^, Sc^3+^.

**Table 2:** Potential chloride removal rate of calcium aluminate-layered double hydroxides (CaAl-LDH) phases obtained by pseudo-second order kinetic model in previous studies [31,67,68].

CaAl-LDH Type	Condition			*Q_e,ca_* * (mg·g^−1^)	*k*_2_ (g·mg^−1^·min^−1^)	*k*_2_*Q_e_*^2^ ** (mg·g^−1^·h^−1^)	Ref.
	Temp. (°C)	pH	Cl Concentration (mol/L)				
CaAl-NO_3_	-	-	0.12–0.48	23.1–25.7	0.0024–0.0030	1.58–1.64	[31]
CaAl-NO_3_	20	12.71	0.005–0.09	-	0.0024–0.0046	-	[67]
CaAl-NO_3_	25	7–13	0.08–0.24	91.844	0.00119	10.038	[68]

* calculated chloride adsorption capacity by means of pseudo-second-order model. ** initial chemisorption rate.

**Table 3:** Potential Chloride removal capacity of CaAl-LDH phases in previous studies [30,67,68].

CaAl-LDH Type	Condition		Capacity (mg/g)	Model	Ref.
	Temp. (°C)	pH			
CaAl-NO_3_	25	-	119.83–159.7	Langmuir	[30]
CaAl-NO_3_	25	7–13	155.5–211.324	Langmuir	[67]
CaAl-NO_3_	20	12.71	118.59	Langmuir	[68]

## Data Availability

Data sharing not applicable.
